# Protection of palmitic acid-mediated lipotoxicity by arachidonic acid via channeling of palmitic acid into triglycerides in C2C12

**DOI:** 10.1186/1423-0127-21-13

**Published:** 2014-02-12

**Authors:** Hyae Gyeong Cheon, Young Sik Cho

**Affiliations:** 1 Department of Pharmacology, Gachon University School of Medicine, Incheon 406-799, South Korea; 2 Gachon Medical Research Institute, Gil Medical Center, Incheon 405-760, South Korea; 3 College of Pharmacy, Keimyung University, 1000 Sindang-dong, Dalseo-gu, Daegu 704-701, South Korea

**Keywords:** Arachidonic acid, Palmitic acid, Lipotoxicity, Insulin resistance, C2C12 myotube, Triglyceride

## Abstract

**Background:**

Excessive saturated fatty acids have been considered to be one of major contributing factors for the dysfunction of skeletal muscle cells as well as pancreatic beta cells, leading to the pathogenesis of type 2 diabetes.

**Results:**

PA induced cell death in a dose dependent manner up to 1.5 mM, but AA protected substantially lipotoxicity caused by PA at even low concentration of 62 μM, at which monounsaturated fatty acids including palmitoleic acid (POA) and oleic acid (OA) did not protect as much as AA did. Induction of cell death by PA was resulted from mitochondrial membrane potential loss, and AA effectively blocked the progression of apoptosis. Furthermore, AA rescued significantly PA-impaired glucose uptake and -signal transduction of Akt in response to insulin.

Based on the observations that polyunsaturated AA generated competently cellular droplets at low concentration within the cytosol of myotubes compared with other monounsaturated fatty acids, and AA-driven lipid droplets were also enhanced in the presence of PA, we hypothesized that incorporation of harmful PA into inert triglyceride (TG) may be responsible for the protective effects of AA against PA-induced lipotoxicity. To address this assumption, C2C12 myotubes were incubated with fluorescent probed-PA analogue 4, 4-difluoro-5, 7-dimethyl-4-boro-3a,4a-diaza-*s*-indacene-3-hexadecanoic acid (BODIPY FL C16) in the presence of AA and their subsequent lipid profiles were analyzed. The analyses of lipids on thin layer chromatograpy (TLC) showed that fluorescent PA analogue was rapidly channeled into AA-driven TG droplets.

**Conclusion:**

Taken together, it is proposed that AA diverts PA into inert TG, therefore reducing the availability of harmful PA into intracellular target molecules.

## Background

Type 2 diabetes is a clinical disease characterized by disruption in the metabolism of glucose and lipids, and consequential failure in the production of insulin as well as insulin resistance [1,2]. These dysfunctions are ascribed partly to a reduced disposal of blood glucose by peripheral tissues such as fat and muscle tissue. In addition, individual with type 2 diabetes shows abnormal lipid dynamics, which seem to be an early events in the development of diabetes [3,4]. The dyslipidemia associated with diabetes is characterized by a high plasma triglyceride concentration, low high-density lipoprotein (HDL)-cholesterol concentration and increased concentration of low-density lipoprotein (LDL)-cholesterol particles [5]. The lipid changes are caused by increased free fatty acid flux secondary to insulin resistance*.* Therefore, clinical trial of multiple lipid-lowering drugs and supplements provides patients with new strategies to reduce lipid levels [6].

Especially, chronic elevation in plasma free fatty acids (FFAs) levels is commonly associated with impaired insulin-mediated glucose uptake in skeletal muscles although the precise mechanism by which FFAs are involved in the development of muscle insulin resistance remains unknown yet. At first, Randle and his co-workers proposed the mechanism for fat-induced insulin resistance, demonstrating that increased fat oxidation is responsible for the insulin resistance associated with obesity [7]. Later, Shulman and colleagues challenged the conventional Randle hypothesis [8]. It has been suggested that the drop in muscle glycogen synthesis is preceded by a decrease in intramuscular glucose-6-phosphate, advising that a surge in the plasma fatty acid concentrations induces insulin resistance by inhibiting glucose uptake or phosphorylation activity. Thereafter, a unifying hypothesis for insulin resistance has been proposed as follows. Namely, an increase in the intracellular fatty acid metabolites lead to initial activation of serine/threonine kinase cascade, followed by phosphorylation of serine/threonine sites on insulin receptor substrates (IRS) [9-12]. The phosphorylation on IRS, in turn, inhibits the recruitment and activation of PI3-kinase, resulting in the decreased activation of glucose transport activity and other downstream events [11]. In fact, the saturated fatty acids palmitate (PA) and stearate (SA), but not their monounsaturated counterparts oleate (OA) and palmitoleate (POA), blocks insulin activation of Akt/PKB with concomitant accumulation of ceramide and diacylglycerol (DAG) in C2C12 myotubes [13]. In contrast, unsaturated fatty acids have distinctive roles in preventing development of fatty acid-induced insulin resistance and diabetes [14]. Mechanistically, triacylglyceride (TG) accumulation by unsaturated fatty acid oleic acid protects against PA-induced lipotoxicity in non-adipose cell Chinese hamster ovary (CHO) [15]. Therefore, fatty acid compositions and their saturation degree are considered to be critical in pathogenesis of diabetes.

Here, we aimed at understanding the protective mechanism of polyunsaturated fatty acid (PUFA) arachidonic acid (AA) against PA-mediated lipotoxicity. AA itself is the precursor of a number of biologically active metabolites formed by cyclooxygenase (COX) and lipoxygenase (LOX) pathways [16]. The eicosanoids such as prostaglandins (PGs) and leukotrienes(LTs), which are respectively produced by COX and LOX activity, play important roles in regulating many physiological processes and acute inflammatory responses [17]. Additionally, an esterified AA is readily converted to phospholipids for membrane bilayer or triglycerides for storage of energy [18]. Thus, to examine the preventive effect of polyunsaturated fatty acid AA on saturated fatty acid-mediated lipotoxicity, skeletal muscle cell C2C12 was supplemented with PA in the presence of AA. For reference, an AA non-metabolic analogue eicosatetraynoic acid (ETYA) was also tested with respect to cytotoxicity, DNA fragmentation and cellular response to insulin to address whether AA metabolism is responsible for its beneficial effects. In our results, AA but not ETYA reversed completely the deleterious effects of PA on C2C12 cells such as cell damage, impaired glucose uptake and insulin signaling pathways.

The preventive potency of AA against PA was not affected by various inhibitors which can block possible metabolic pathways of AA. The substantial ability of AA to generate intracellular lipid droplets raised the possibility that inert TG might be a reservoir of sequestrating harmful saturated FA, limiting lipotoxic PA accessible to cells. In fact, incubation of C2C12 in media supplemented with PA and AA displayed more Nile-red (9-diethylamino-5H-benzo[alpha]phenoxazine-5-one)-positive cells than that in media containing PA and monounsaturated fatty acid. Accordingly, to verify the incorporation of PA into TG, fluorescent PA analogue 4, 4-difluoro-5, 7-dimethyl-4-boro-3a, 4a-diaza-s-indacene-3-hexadecanoic acid (BODIPY FL C16) was employed to trace trafficking of the PA. Fluorescent PA analogue was localized in AA-driven intracellular lipid droplets, and was also found in TG fraction resolved on thin layer chromatography (TLC), indicating that PA can facilitate TG synthesis, and be directly incorporated as fatty acid constituent of TG.

Taken together, we propose that AA protects PA-caused lipotoxicity via distribution of it into inert TG, leading to lowering of harmful free fatty acids accessible to cells.

## Methods

### Materials

C2C12 cells (mouse skeletal muscle cell lines: ATCC CRL-1772) was obtained from the American Type Culture Collection (Rockville, MD, USA). Dulbecco's Modified Eagle's Medium (DMEM), Dulbecco’s phosphate buffered saline (D-PBS), fetal bovine serum and antibiotics were purchased from Gibco (Grand Island, NY, USA). 5, 5’, 6, 6’-tetrachloro-1, 1’, 3, 3’-tetraethylbenzimidazolcarbocyanine iodide (JC-1) and BODIPY FL C16 fatty acid were from Molecular Probes (Leiden, The Netherlands). 2-[1,2-^3^H]-deoxy-D-glucose was from Amersham Biosciences (Piscataway, NJ. USA). Eicosatetraynoic acid (ETYA) was from Calbiochem (San Diego, CA, USA). DNA purification system and cytotoxicity detection kit (lactate dehydrogenase, LDH) were available from Gentra systems (Minneapolis, MN, USA) and Roche Diagnostics (Indianapolis Mannheim, Germany), respectively. The anti-phospho-Akt-ser473 and anti-Akt antibodies were from Cell Signaling Technology (Beverly, MA, USA). Nile red, lipid standards and all other chemicals were purchased from Sigma (St. Louis, Mo, USA).

### Cell culture

C2Cl2 cells were grown and maintained in DMEM supplemented with 10% fetal bovine serum and 1% antibiotics (100 U/ml penicillin and 100 μg/ml streptomycin) in 5% CO_2_ environment, and then differentiated into myotubes by shifting growth media into differentiation media containing 2% horse serum and allowing to incubate for at least 4 days. For all the cytotoxic assays, unless otherwise indicated, cells were plated into 96-well plates at 1 × 10^4^/well and allowed to grow for 24 h at 37°C.

### Preparation of fatty acid-BSA complex

The fatty acid-BSA complex used in these experiments was prepared according to the literature [19]*.* In brief, different kinds of fatty acids were dissolved in ethanol:H_2_O (1:1, vol:vol) at 50°C at a final concentration of 150 mmol/l. Aliquots of stock solutions were complexed with fatty acid-free BSA (10% solution in H_2_O) by stirring for 1 h at 37°C and then diluted in culture media in order to adjust the final molar ratio of fatty acid:BSA at 5:1 and ethanol concentration at less than 0.33% (vol/vol).

### Determination of LDH release for cell viability

To test whether FFAs have toxic effects on C2C12 cells, we measured lactate dehydrogenase (LDH) activity in the culture media. After exposure of myotubes to FFAs for 24 h, supernatant aliquots were obtained for quantification of LDH released into the medium, using a colorimetric end-point procedure. The absorbance was measured at 492 nm using a plate Reader. Reference controls for 0 and 100% cytolysis were BSA-containing medium alone or medium containing 0.1% (v/v) Triton X-100, respectively. All assays were carried out in triplicate.

### DNA fragmentation

For quantitative determination of apoptotic DNA fragmentation, total DNA was extracted from fatty acid-treated cells using a DNA purification kit. The DNA fragmentation induced by PA was analyzed on ethidium bromide (EtBr)-stained 1.5% agarose gels.

### Measurement of mitochondrial membrane potential

Mitochondrial membrane potential was determined by JC-1 fluorescence. JC-1 is a cationic dye that exhibits potential-dependent accumulation in mitochondria, as indicated by a fluorescence emission shift from green (~525 nm) to red (~590 nm), making it useful for ratiometric measurements. Once membrane potential changes to −100 mV, JC-1 exists as green monomers with emission peak around 525 nm whereas JC-1 forms aggregates and its emission shifts towards 590 nm as the membrane is hyperpolarized (−140 mV). To monitor the mitochondrial potential caused by fatty acids, cells were incubated in D-PBS containing 1 g/L glucose and 10 μM JC-1 for 10 min at 37°C. Thereafter, cells were washed once, and intensities were measured at paired excitation and emission wavelengths of 485/530 and 530/590 for green fluorescence and red fluorescence, respectively, with a Synergy HT (BioTek Instruments, Winooski, VT, USA) plate reader. The ratio of red to green fluorescence was indicated for the measurement of the mitochondrial membrane potential. All compounds were dissolved in DMSO stock solution so that the final DMSO levels did not exceed 1%.

### Effects of fatty acids on insulin-stimulated glucose uptake and Akt phosphorylation

Terminally differentiated myotubes (C2C12) were exposed to increasing concentrations of PA or PA along with either AA or ETYA for 24 h. FAs-exposed cells were rinsed and subjected to glucose uptake assay in response to 100 nM insulin in Krebs Ringer phosphate (KRP) buffer containing 0.5 μCi/well of [^3^H]-2-deoxy-glucose supplemented with 10 μM 2-deoxyglucose. Glucose uptake was assessed as an increase of disintegrations per minute (DPM)/well in response to insulin stimulation versus basal glucose uptake for 30 min at 37°C. After washing with KRP buffer twice, cells were air-dried, solubilized with 500 μl of 0.1 N NaOH for 2 h, and resulting 400 μl lysate was mixed with 40 μl of 1 N HCl to neutralize it and 4 ml scintillation cocktail solution added, and then the radioactivities were counted.

For signaling pathway from insulin-insulin receptor (IR) through its downstream protein Akt, myotubes were seeded at a density of 5*10^5^ into 6 well plates. After cell attachment, cells were treated with 0.5 mM PA along with AA at 31 or 62 μM and ETYA at 31 μM for 24 h, respectively. Cells were rinsed with D-PBS, stimulated with 100 nM insulin for 30 min and then lysed in lysis buffer containing 0.5% Triton X-100, 1 mM EDTA and protease inhibitors. The consequential cell lysates were subjected to 10% SDS-PAGE and blotting onto polyvinylidene difluoride (PVDF) membrane for detection of pAkt an Akt with antibodies.

### Effects of AA on TG contents by Nile red staining

Myotubes at a density of 5*10^5^ were exposed to 0.5 mM PA together with unsaturated fatty acids at a concentration of 62.5 μM for 24 h. Treated cells were rinsed with PBS and fixed with 10% formaldehyde solution and finally stained with Nile red (9-diethylamino-5*H*-benzo[α]phenoxazine-5-one) in a final concentration of 1 μg/ml. After the plates were incubated for 10–30 min at 4°C and then washed three times with PBS, cellular Nile red-stained lipid droplets were observed by using fluorescence microscopy. In addition, fluorescence of cells stained with Nile red was measured at room temperature with excitation and emission at 530 nm and 590 nm, respectively. The fluorescence intensities correspond to the fatty acids-driven neutral lipids.

### Incorporation of BODIPY FL C16 into AA-driven TG and TLC analysis of lipid extracted from C2C12

The growing cells were supplemented with either 7.5 μM of fluorescent PA analogue BODIPY FL C16 alone or 7.5 μM of BODIPY C16 plus 62.5 μM AA. Twenty four hours later, cells were washed with PBS, fixed with 10% formaldehyde solution and then observed for the incorporation of PA analogue into AA-driven lipid droplets using fluorescence microscopy. For lipid analyses on TLC, total lipids were extracted from cells according to Folch method [20]. In brief, cell pellets were homogenized in chloroform:methanol mixture (2:1), and then resulting extracts were washed by addition of 0.2 volume of PBS to eliminate the non-lipid contaminants. The final lower phase containing total lipids were spotted on the TLC, and developed in solvent of hexane: diethyether: acetic acid (70:30:1, V/V). Plate were dried in air and illuminated under long wave UV to visualize the fluorescent probe.

### Statistical analyses

Results were expressed as means ± S.E.M. Statistical significance was evaluated using Student’s *t*-test, and *p* <0.05 was considered as statistically significant.

## Results

### Cytotoxic activity of PA and protection of AA from PA-caused cytotoxicity in C2C12

**Figure 1:**
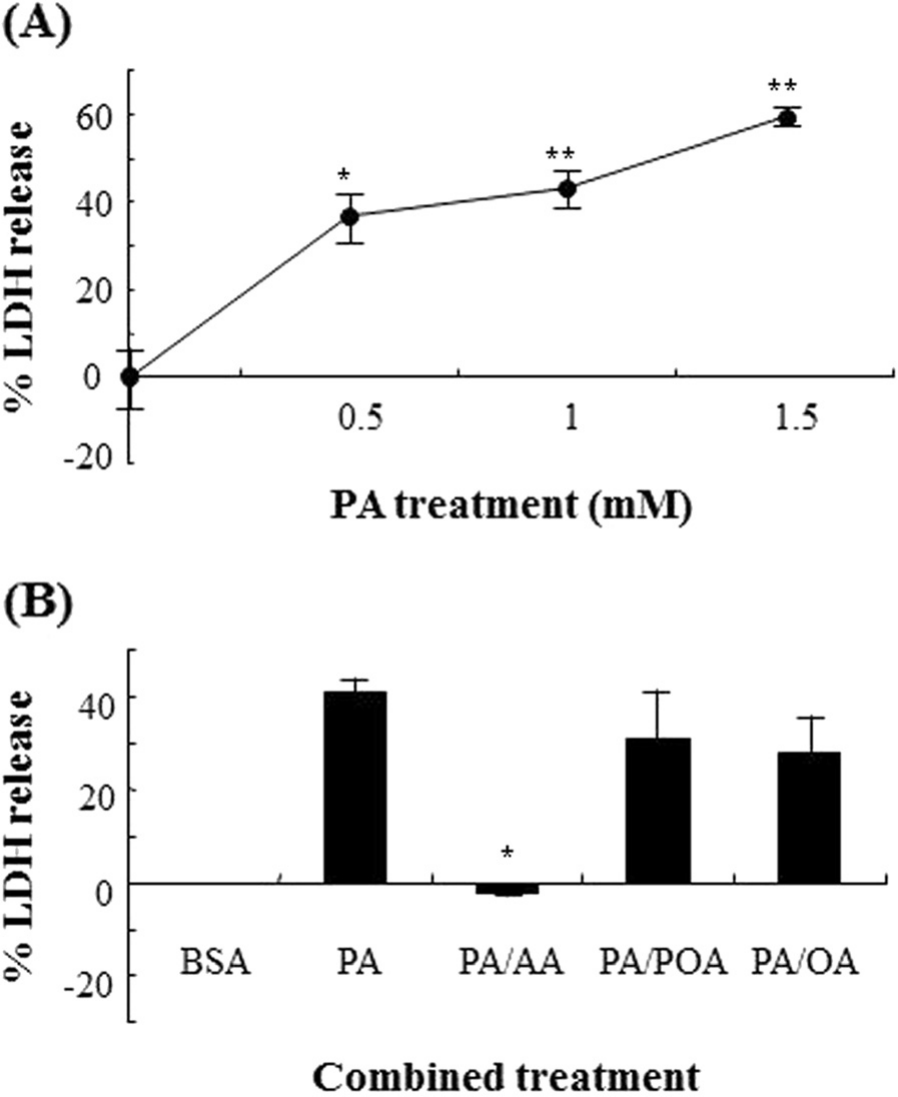
**Lipotoxicity of saturated fatty PA as a function of concentration (A) and the effect of unsaturated fatty acids on PA-mediated lipotoxicity (B).** Cells grown at 2*10^4^ were treated with increasing concentrations of PA for 24 h. To examine the protective effects of unsaturated fatty acids on lipotoxicity of PA, cells were coincubated with 0. 5 mM PA and 62.5 μM unsaturated fatty acids indicated for 24 h. LDH release into media was assayed for the evaluation of lipotoxicity mediated by supplementation of fatty acids, and relative LDH release for each group was calculated from absorbance values being set sample completely lysed in Triton X-100 and culture medium left untreated to 100% and 0%, respectively.

As a preliminary test, myotubes were exposed to PA at different concentrations to understand the titration effects of PA on cell death. When cellular toxicity was measured by LDH released from dead cells, PA caused substantially cell death in a dose dependent manner over 0.5-1.5 mM concentrations (Figure 1A). However, AA alone but not monounsaturated fatty acid significantly protected cells from PA lipotoxicity at relatively low concentration of unsaturated fatty acids, although other mono unsaturated fatty acids had weak protective effects against PA toxicity with no statistical significance (Figure 1B).

### Protective effects of AA on PA-induced DNA ladder and –mitochondrial membrane potential

**Figure 2:**
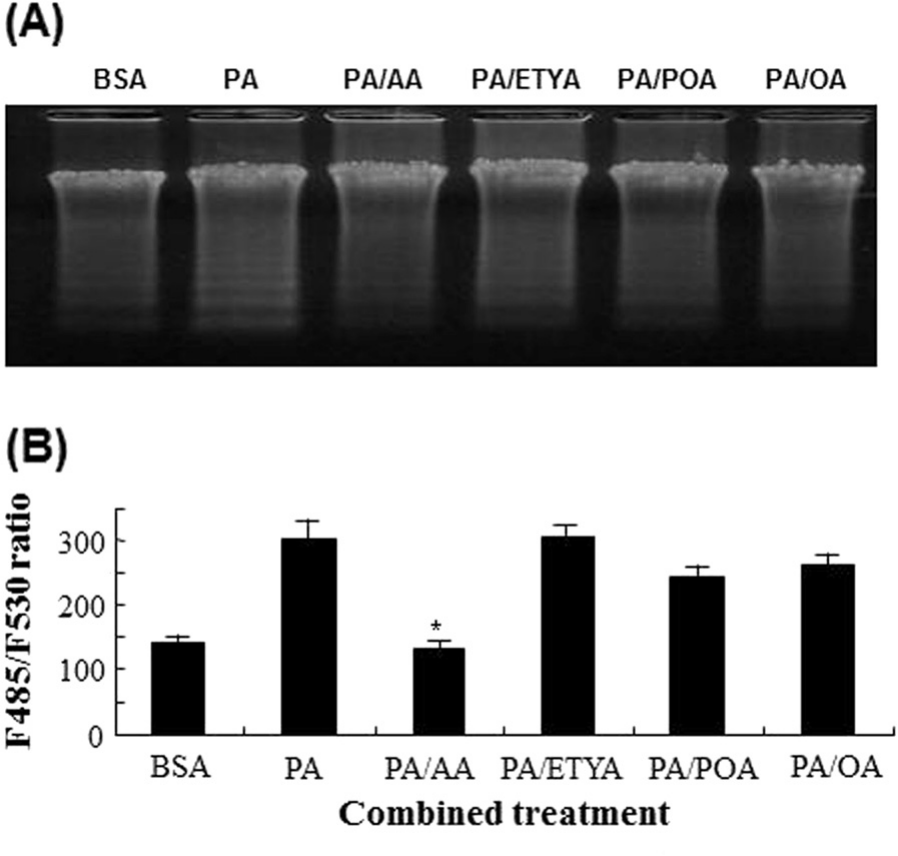
**The changes in DNA damage (A) and mitochondrial membrane potential (B) when C2C12 cells were treated with either PA alone or PA and unsaturated fatty acids.** Cells at a density of 5*10^5^ were exposed to PA in combination with unsaturated fatty acids for 24 h. The genomic DNAs from fatty acids-treated myotubes were isolated and resolved on 1.5% agarose electrophoresis. For the measurement of mitochondrial membrane potential, myotubes were overloaded with 0.5 mM PA and unsaturated fatty acids at a concentration of 62.5 μM for 24 hr. The fatty acids-treated cells were rinsed with phosphate-buffered saline (PBS), incubated with culture media containing 5 μM JC-1 for 30 min and subsequently washed twice with PBS. Fluorescent ratios were calculated from fluorescence measured at 485/545 and 530/590 nm (excitation/emission), respectively. Each bar represents the mean of 4 separate experiments ± S.E.M. *p < 0.05 versus PA-treated group.

In the previous articles observed in other cell types, PA has been reported to induce cell death showing typical apoptotic indications like DNA laddering and DNA condensation. To simply examine whether PA-mediated cell death is associated with apoptosis, DNA laddering, an indicative of apoptotic cell death, was checked after cells were loaded with PA or with PA and unsaturated fatty acids (Figure 2A). Treatment of C2C12 with PA induced obviously DNA cleavage, and AA but not ETYA prevented more effectively PA-caused DNA laddering than did POA or OA. Apoptosis is associated with a wide set of biochemical and physical changes in cytoplasm, nucleus and plasma membrane. However, the alteration in the mitochondrial permeability transition precedes cellular apoptosis, that is, mitochondrial opening induces depolarization of the transmembrane potential with concomitant release of apoptogenic factors and loss of oxidative phosphorylation. In this presentation, changes in mitochondrial potential of C2C12 cells exposed to PA were measured by using JC-1 (Figure 2B). As apoptotic progression undergoes, the electrochemical gradient across the mitochondrial membrane collapses and is aptly monitored by JC-1 dye. PA caused a decrease in mitochondrial membrane potential, which was indicated by the increased ratio of fluorescence (485 nm/530 nm). Furthermore, PA-induced mitochondrial dysfunction was reversed to the control level by AA. In contrast, ETYA did not produce noticeable change in mitochondrial potential loss caused by PA while POA and OA partially restored it.

### Effects of AA on insulin-stimulated glucose uptake and –mediated signal transduction in C2C12 cells

**Figure 3:**
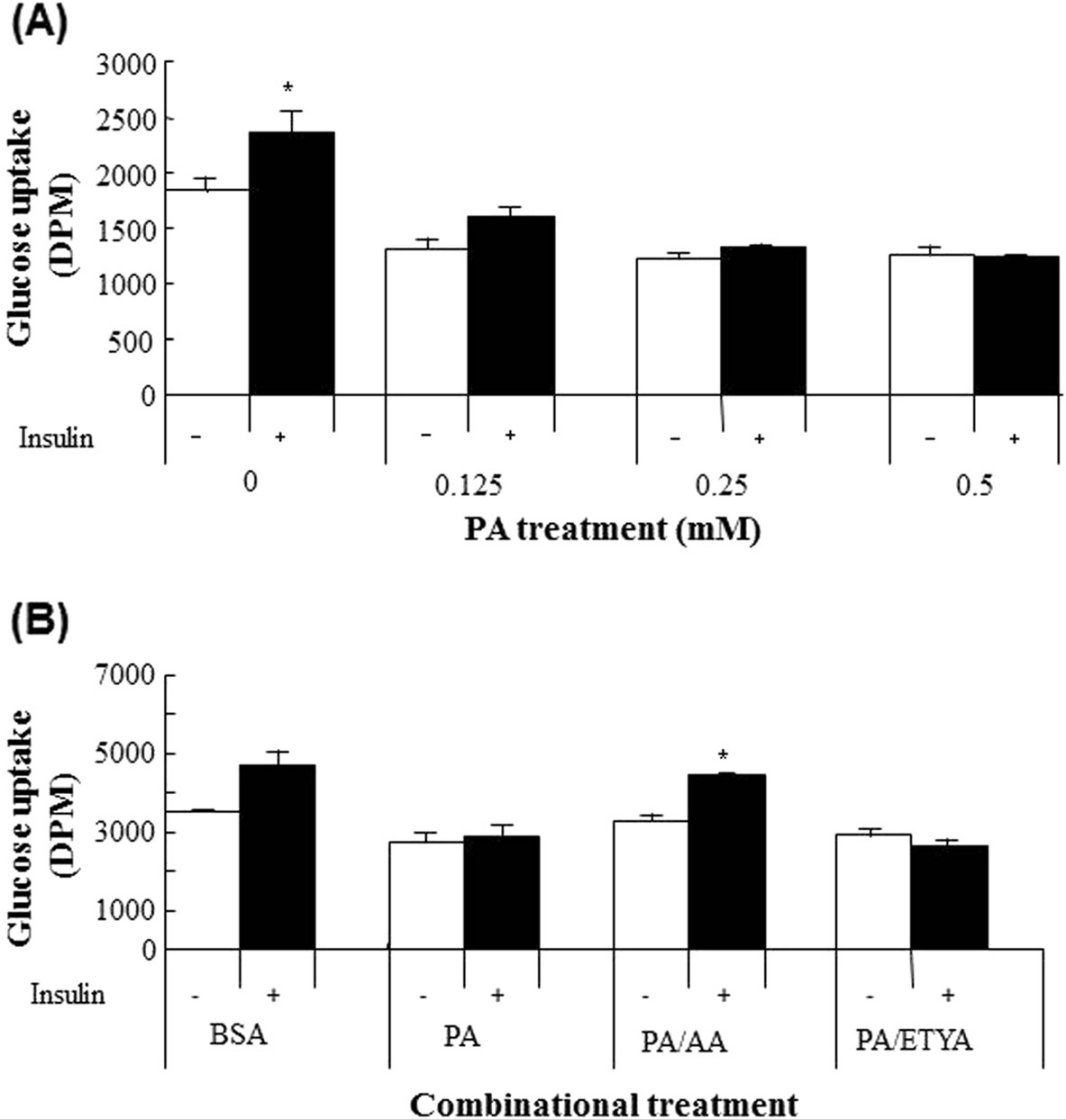
**The dose dependent impairment of insulin-stimulated glucose uptake in C2C12 treated with PA (A) and improvement of glucose uptake in response to insulin by AA (B).** Differentiated myotubes C2C12 cells were exposed to increasing concentrations of PA or 0.5 mM PA along with either AA or ETYA at the concentration of 62.5 μM for 24 h. Subsequently, cells were rinsed and subjected to glucose uptake assay in response to 100 nM insulin in a buffer containing 10 μM 2-deoxyglucose. Glucose uptake was expressed in an increase of DPM/well in response to insulin stimulation versus basal glucose uptake. *p <0.05 versus basal glucose uptake.

**Figure 4:**
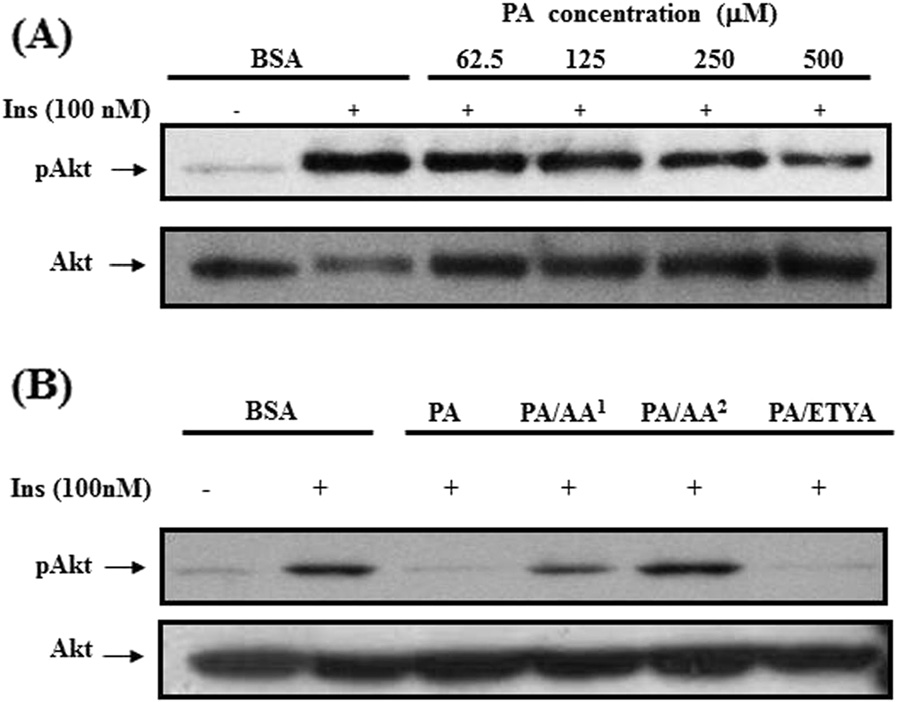
**Insensitization of insulin signal pathways when supplemented with different concentrations of PA (A) and the restoration of PA-abrogated insulin signal pathway in the presence of AA (B).** To optimize insensitization condition of insulin signal transduction by PA, PA in 2-fold dilution was added to myotubes culture and kept for 24 h under CO_2_ incubator. In addition, to test the preventive effect of AA against insulin signal transduction impaired by PA, cells were treated with either 0.5 mM PA plus two doses of AA^1^ and AA^2^ (31 and 62.5 μM) or 0.5 mM PA plus 62.5 μM ETYA for 24 h, respectively. Fatty acids-exposed cells were rinsed with PBS, stimulated with 100 nM insulin for 30 min and then lysed in lysis buffer containing 0.5% Triton X-100, 1 mM EDTA and protease inhibitors. The prepared cell lysates were subjected to 10% SDS-PAGE and blotting onto PVDF membrane for immunoblotting against pAkt an Akt using specific antibodies.

In C2C12 cells, insulin treatment alone (100 nM) enhanced glucose uptake by about 30%. However, PA exposure lowered basal glucose uptake, and abolished insulin-stimulated glucose uptake in a dose dependent manner as well (Figure 3A). In contrast, supplementation of myotubes with AA but not ETYA reversed significantly cellular insensitization to insulin for glucose uptake (Figure 3B). To further understand the underlying signaling pathways associated with glucose uptake, Akt phosphorylation was examined upon stimulated with insulin in the presence of either PA or PA and AA (Figure 4A and 4B). Treatment of cells with PA inhibited enhancement of Akt phosphorylation in response to insulin. Similar to the results of glucose uptake, AA but not ETYA enabled cells to restore signaling pathway, fostering insulin actions.

### Effects of AA on TG accumulation

**Figure 5:**
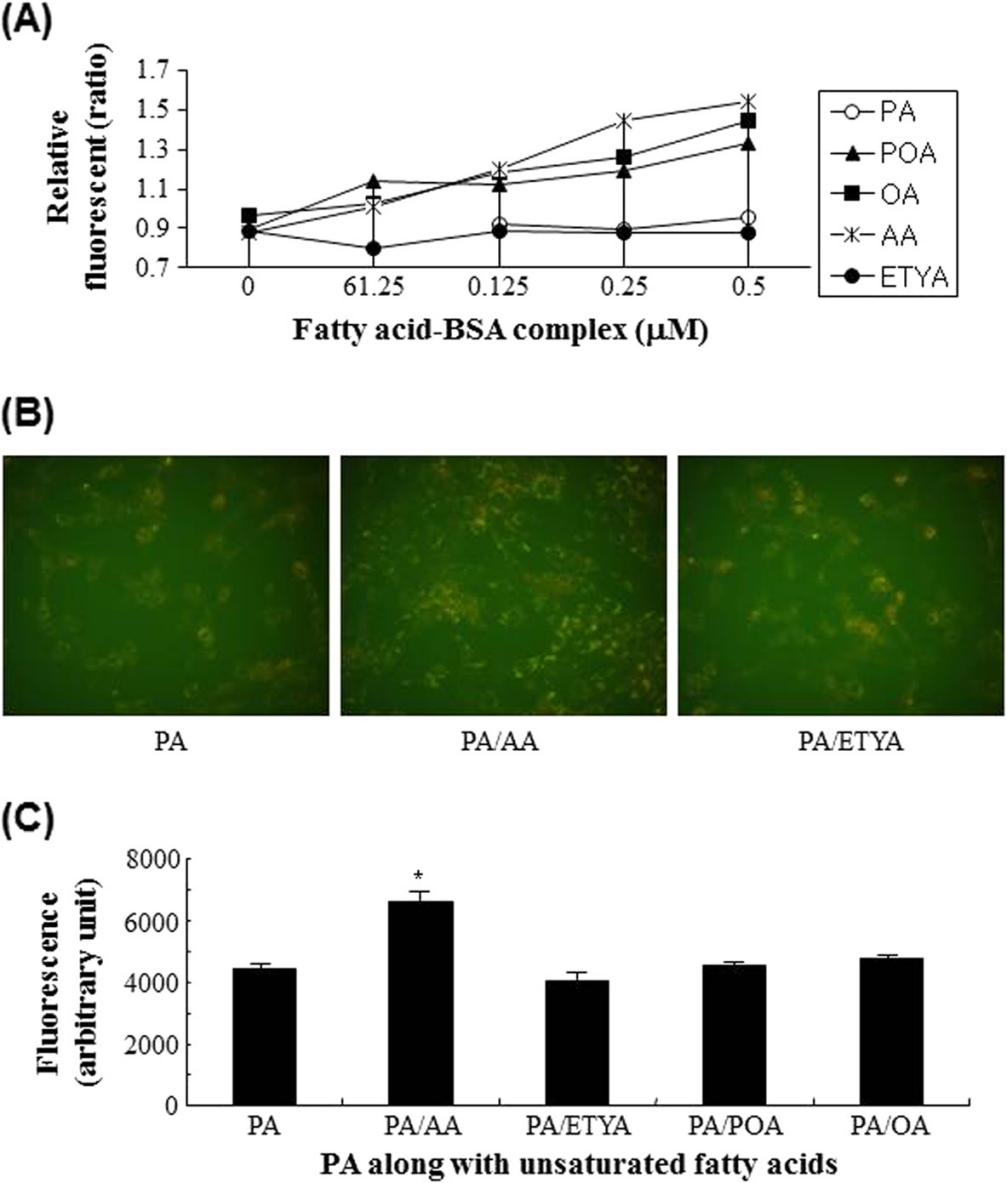
**Augmentation of PA-driven lipid droplets by AA loads in C2C12 cells.** The driving potency of each fatty acids to produce lipid droplets during C2C12 culture was measured by Nile red staining of cells grown in media containing various doses of fatty acids-BSA complex **(A)**. Also, images of lipid droplets within cytosol were taken when cells were overloaded with 0. 5 mM PA alone (left), 0.5 mM PA plus 62.5 μM AA (middle) or 0.5 mM PA plus 62.5 μM ETYA (right) **(B)**. To quantitate stimulated incorporation of PA into lipid droplets by unsaturated fatty acids, PA in combination with unsaturated fatty acids at 62.5 μM were loaded into C2C12 cell culture and allowed to incubate for 24 h. Relative fluorescence of neutral droplets were measured following Nile-red staining and fixation of them **(C)**. *p < 0.05 versus PA-treated group.

When cells were treated with unsaturated fatty acids alone, lipid droplets was observed in the cytosol within cells. Therefore, the relative formation of intracellular neutral lipid droplets driven by each fatty acid was measured by Nile red staining (Figure 5A). Exposure of cells to PA showed little neutral lipid droplets while unsaturated fatty acids did obviously in a dose dependent way. Microscopic observation showed that PA strengthen Nile red staining of lipid droplets in cells overloaded with AA, whereas ETYA did not so much (Figure 5B). To corroborate the augmentation of PA-driven TG synthesis in the presence of AA, quantitative analyses of TG were also performed in C2C12 cells supplemented with different combinations of fatty acids (Figure 5C). Only AA exhibited strong Nile red staining together with PA at such a low concentration compared with other unsaturated fatty acids, demonstrating that AA at a low dose stimulated effectively incorporation of PA into TG.

**Figure 6:**
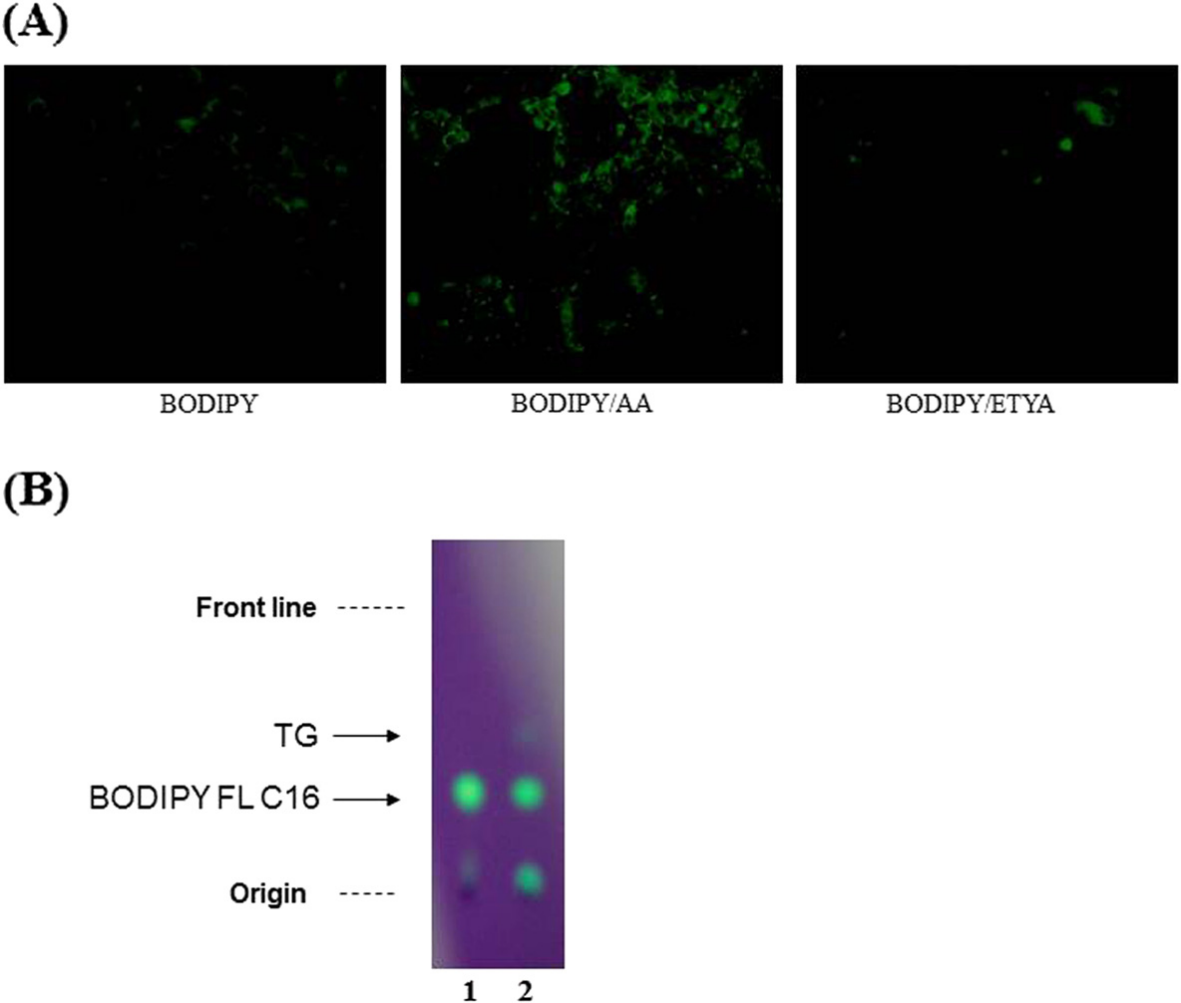
**The incorporation of PA into AA-driven neutral lipid TG.** Microscopic images showing localization of fluorescent PA analogue BODIPY FL C16 within lipid droplets of C2C12 cells exposed to AA **(A)** and lipid analyses extracted from C2C12 cells on TLC **(B)**. To examine the trafficking of PA into AA-driven TG, the myotubes were supplemented with either 7.5 μM of fluorescent PA analogue 7.5 μM BODIPY FL C16 alone or 7.5 μM of BODIPY FL C16 plus 62.5 μM AA or 62.5 μM ETYA for 24 h. Cells were washed with PBS, fixed with 10% formaldehyde solution and then observed for the incorporation of PA analogue into AA-driven TG using fluorescence microscopy equipped with a B filter set. For lipid analysis, total lipids were extracted from cells treated under the same condition as in above. Lipids on TLC were developed and visualized as described in Methods. Lane 1 and 2 were, respectively, loaded with lipids extracted from cells left treated with BODIPY FL C16 or BODIPY FL C16 plus AA.

To prove the incorporation of PA into neutral lipid droplets, fluorescent PA analogue BODIPY FL C16 was employed (Figure 6A and 6B). BODIPY FL C16 across cell membrane was faintly distributed within entire cytosol around nuclei, whereas fluorescent BODIPY FL C16 was mostly observed in refractory droplets. EYTA was ineffective in facilitating the incorporation of PA into lipid droplets (Figure 6A). To support that PA is incorporated in TG of AA, lipid fractions extracted from the cells were analyzed on the TLC. The fluorescent PA analogue itself reduced with AA overloads, and conversely, TG spot with fluorescence appeared freshly in the presence of AA (Figure 6B).

## Discussion

In spite of accumulating evidences that circulating FFAs have been linked to Type II diabetes, underlying mechanisms as to how FFAs lead to lipotoxicity remain unknown. In the present study, exposure to PA caused substantial cytotoxicity in a concentration-dependent manner up to 0.5 mM. The cell death mediated by PA appeared to be derived from apoptosis as revealed from DNA fragmentation, an apoptosis indicator. FFA-induced apoptosis has been previously reported in pancreatic β-cells [21], hepatocytes [22], brain tumors [23] and skeletal muscle [24] but the details of FFA-induced apoptosis has not still been understood. Moreover, it has been still obscure even whether FFA-induced apoptosis is executed by direct effects of FFAs or indirect effects through its metabolites. However, it seems to be apparent that mitochondrial dysfunction is closely associated with FFA-induced apoptosis*.* In another article, long chain FFAs including PA and OA not only increases O_2_ consumption but also reduces mitochondrial membrane potential [25]. In our data, PA was shown to induce apoptosis via alteration in mitochondrial potential. The depolarized mitochondrial potential, an early event in the process of apoptosis, occurred in the presence of PA and was significantly restored by AA but not monounsaturated fatty acids. On the contrary, there has recently been a growing body of reports that PA induces predominantly alternative cell deaths like autophagy and necrosis [26-28]. PA, but not OA, leads to an increase in autophagic flux via protein kinase C activation. In addition, PA causes lipotoxic cell death of endothelia cells in a necroptotic manner. More importantly, its lipotoxicity is rescued by pharmacological inhibitor and genetic knockdown. Accordingly, PA induces wide spectra of cell death types, depending cell types and cellular context, so that strategies tailored to death modes should be taken to counteract the lipotoxicity signaling derived from PA.

It is generally proposed that mitochondrial (mt) dysfunction and subsequent apoptotic cell death are mediated by PA-generated ROS [29,30]. It remains controversial for ROS generation to be dependent on ceramide synthesis. It was previously reported that palmitate induced mtROS generation through the de novo synthesis of ceramide, accumulation of which leads to the apoptosis in L6 myotubes [31]. Conversely, it was suggested that PA caused apoptosis via ROS generation independent of ceramide formation [15]. However, since PA alone in our result did not cause ROS production, ROS did not seem to link directly to PA-induced lipotoxicity in C2C12. Consistent with this observation, there is an article that ROS are not involved in the apoptosis induction of neonatal cardiomyocytes by PA [32]. More notably, PA/Ca(2^+^) complex has been proposed to induce directly the opening of mitochondrial cyclosporin A (CsA)-insensitive pore [33].

Interestingly, unsaturated fatty acids have been known to exert antagonizing effects against fatty acid-induced lipotoxicity. Treatment of islet cells with unsaturated fatty acids prevents completely saturated FA-induced apoptosis of human β-cells [34]. More apparently, saturated FFAs induce apoptosis in granulose cells whereas AA antagonizes saturated FFA-induced apoptosis [35]. Recently, it has been highlighted that AA protects more effectively β-cells from PA-induced lipotoxicity than monounsaturated fatty acids [36]. In agreement with previous reports, we revealed that unsaturated fatty acids rescued PA-induced lipotoxicity including cytotoxicity and insulin signal. As a result, the preventive potency of unsaturated fatty acids against PA was in the decreasing order of AA > POA = OA > ETYA. Unlike other unsaturated fatty acids, a non-metabolizable triple-bond analogue of AA ETYA did not block lipotoxicity-induced biological malfunction, therefore indicating that esterification of unsaturated fatty acids is required for its antagonizing effect on PA-induced lipotoxicity.

From the microscopic observation that all the unsaturated fatty acids except for ETYA used in this experiment could generate discrete lipid droplets in the cytosol of C2C12, as visualized by Nile-red staining, we raised the possibility that unsaturated property of fatty acids might drive the incorporation of PA into TG, inert neutral lipids. PA alone did not generate a large number of lipid droplets, but it enhanced AA-derived TG formation in a concentration-dependent manner. The biological effect of TG accumulation on PA-induced lipotoxicity seems still controversial. Traditionally, TG accumulation is thought to be indicative of dangerous signal in the development of pathogenesis associated with disordered lipid metabolism. TG accumulation in pancreatic β-cells has been closely associated with impairment in insulin secretion to glucose [37]. Moreover, it was claimed that lipotoxicity of β-cells was associated with glucose-dependent esterification of fatty acids into neutral lipids [38]. On the contrary, it has been published that unsaturated fatty acids with high ability to synthesize TG are implicated in the prevention of PA-mediated apoptosis by sequestrating deleterious PA into TG storage form [15]. Also, TG has been proposed to play a critical role in trafficking and modulation of free fatty acids [39]. In our experiments, neutral lipid mass was augmented upon coincubation of PA with arachidonic acid (AA), accompanied with a decrease in PA-induced lipotoxicity. Cnop et al., has demonstrated that an inverse correlation is observed between the percentage of dead β-cells and their cellular TG contents [40]. It was further demonstrated that exogenously or endogenously generated unsaturated fatty acids rescued PA-induced apoptosis in non-adipose cells by promoting PA incorporation into TG, accumulation of which represents an initial cellular defense against lipotoxicity in response to acute lipid overload [41]. More recently, AA-induced TG accumulation could protect pancreatic β cells from PA-induced lipotoxicity [36]. There is an interesting report that AA has beneficial effects on a clonal pancreatic β-cell line inflicted with PA via down-regulation of inducible NO synthase (iNOS), the p65 subunit of nuclear factor κB (NF-κB) and p47 subunit of NADPH oxidase [42].

Besides protective effects of unsaturated fatty acids against saturated FA load via TG accumulation, it cannot be ruled out that there may be other mechanisms responsible for the beneficial effects of AA against PA-induced lipotoxicity. The potent unsaturated fatty acid AA itself is readily metabolized into lipid second messengers such as leukotrienes and prostaglandins. To assess whether AA metabolites could confer the protective action against PA-induced lipotoxicity or what sort of AA metabolites could contribute to their protection, specific inhibitors interfering with 3 different pathways for AA metabolism could be employed. No inhibitors affected the protective effects of AA against PA load (data not shown), thereby suggesting that AA-CoA but not AA metabolites is essential metabolites for protection of AA against PA-mediated lipotoxicity. Secondly, cAMP accumulation is suggested to reverse PA-induced apoptosis via both protein kinase A- and cAMP-guanine nucleotide exchange factor-dependent pathways in β-cells [43]. However, either cAMP-generating agents or non-metabolizable cAMP analogue bromo-cAMP did not exert significantly protective effects on the PA-inflicted cell damage (data not shown). Thirdly, the cytotoxic effects of PA might be derived from its physicochemical property. A relatively large surge in partitioning of PA into the phospholipid membrane lowers the membrane fluidity due to high melting temperature (T_M_) [44]. In contrast, high contents of polyunsaturated fatty acids in phospholipid fraction are expected to renders PA-caused rigid membrane flexible due to a drop in transition temperature. The investigation of fatty acid composition and ratio of fatty acid species in plasma membranes will be helpful for further understanding the beneficial roles of AA on membranes overloaded with deleterious PA.

## Conclusions

**Figure 7:**
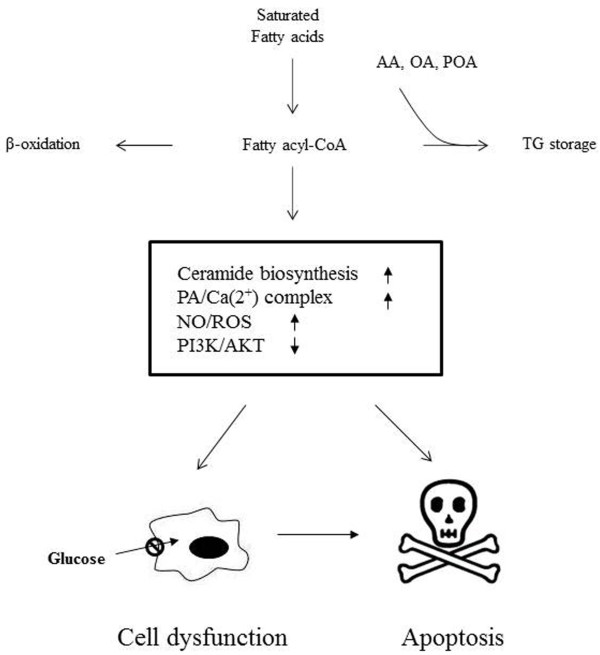
Schematic summary demonstrating the protective effects of AA against PA-mediated lipotoxicity.

Our findings that AA can nullify the deleterious effects of PA via sequestration of PA into TG are recapitulated in the schematic summary (Figure 7). In a scheme, saturated fatty acids are intracellularly activated to produce fatty acyl-CoA, which is further metabolized into β-oxidation or TG accumulation. Overload of saturated fatty acids induces lipotoxicity to promote cell dysfunction and further cell death via generation of ROS, ceramide and modulation of associated molecules. Cotreatment of cells with PA and unsaturated fatty acids diverts excess of saturated fatty acids into inert TG storage form, linking TG production to its protective effect against PA lipotoxicity. Out of unsaturated fatty acids, AA at low levels effectively protected cells from palmitic acid-induced lipotoxicity.

Taken together, we found that AA prevented effectively PA-induced lipotoxicity in myotubes. Primary mechanism by which AA protects myotubes from PA lipotoxicity appears to be converting harmful PA into TG, and its substantial processes are likely to be mediated by AA-CoA, but not AA metabolites. The detailed protective mechanism of AA against PA-induced lipotoxicity will be further elucidated through analysis of lipid compartmentalization and lipid compositions.

## Abbreviations

PA: Palmitic acid; POA: Palmitoleic acid; OA: Oleic acid; AA: Arachidonic acid; ETYA: Eicosatetraynoic acid; TG: Triglyceride; LDH: Lactate dehydrogenase.

## Competing interests

The author declares no directly or indirectly competing interests.

## Authors’ contributions

HGC designed the whole experimental scheme and carried out the experiments. YSC contributed to the study concept, research design, data interpretation and manuscript writing. Both authors read and approved the final version of the manuscript.
